# A Deep Learning Methodology for the Detection of Abnormal Parathyroid Glands via Scintigraphy with ^99m^Tc-Sestamibi

**DOI:** 10.3390/diseases10030056

**Published:** 2022-08-23

**Authors:** Ioannis D. Apostolopoulos, Nikolaos D. Papathanasiou, Dimitris J. Apostolopoulos

**Affiliations:** 1 Department of Medical Physics, School of Medicine, University of Patras, GR 265-00 Patras, Greece; 2 Department of Nuclear Medicine, School of Medicine, University of Patras, GR 265-00 Patras, Greece

**Keywords:** deep learning, parathyroid gland, scintigraphy, artificial intelligence

## Abstract

Background: Parathyroid proliferative disorder encompasses a wide spectrum of diseases, including parathyroid adenoma (PTA), parathyroid hyperplasia, and parathyroid carcinoma. Imaging modalities that deliver their results preoperatively help in the localisation of parathyroid glands (PGs) and assist in surgery. Artificial intelligence and, more specifically, image detection methods, can assist medical experts and reduce the workload in their everyday routine. Methods: The present study employs an innovative CNN topology called ParaNet, to analyse early MIBI, late MIBI, and TcO4 thyroid scan images simultaneously to perform first-level discrimination between patients with abnormal PGs (aPG) and patients with normal PGs (nPG). The study includes 632 parathyroid scans. Results: ParaNet exhibits a top performance, reaching an accuracy of 96.56% in distinguishing between aPG and nPG scans. Its sensitivity and specificity are 96.38% and 97.02%, respectively. PPV and NPV values are 98.76% and 91.57%, respectively. Conclusions: The proposed network is the first to introduce the automatic discrimination of PG and nPG scans acquired by scintigraphy with ^99m^Tc-sestamibi (MIBI). This methodology could be applied to the everyday routine of medics for real-time evaluation or educational purposes.

## 1. Introduction

Parathyroid proliferative disorder encompasses a wide spectrum of diseases, including parathyroid adenoma (PTA), parathyroid hyperplasia, and parathyroid carcinoma [1]. Approximately 83% of primary hyperparathyroidism HPPT is caused by a parathyroid adenoma, followed by primary parathyroid hyperplasia at 15%, and parathyroid carcinoma at 1% or less [2].

End-stage renal failure causes secondary HPPT. In this case, all parathyroid glands (PG) are each enlarged to different degrees. Tertiary HPPT is an unusual situation in which HPPT persists after successful renal transplantation. Lowering serum calcium and parathyroid hormone levels is achieved by calcimimetic drugs. However, HPPT remedy is fully achieved by the surgical excision of abnormal PGs. Imaging modalities that deliver their results preoperatively help localising of PGs and assist surgery. Such methods include neck ultrasound (U/S), parathyroid scintigraphy, dynamic contrast-enhanced computerised tomography, also called 4-D CT, and magnetic resonance imaging (MRI).

The Department of Nuclear Medicine in the University Hospital of Patras, Greece, has specialised in performing parathyroid scintigraphy examinations for more than 20 years [3]. Parathyroid scintigraphy is performed via the intravenous injection of the radioactive tracer ^99m^Tc-sestamibi (MIBI). The dual-phase technique includes the acquisition of early (10 min post-MIBI administration) and late (2 h post-injection) images of the neck and the mediastinum. In early images, MIBI uptake by the thyroid gland may obscure the detection of an underlying parathyroid adenoma. However, most abnormal PGs exhibit prolonged tracer retention and appear prominent in late images, while MIBI clears more rapidly from the thyroid gland. Fast clearance of MIBI from some parathyroid adenomas and many hyperplastic glands is a common cause of false-negative scans. The thyroid subtraction technique requires the administration of a second radioactive tracer (^123^I or ^99m^Tc-pertechnetate) to delineate the thyroid gland. Then, the thyroid image is subtracted digitally from the early MIBI image. Early MIBI uptake by some thyroid nodules is a common cause of false-positive findings. The two techniques can be applied alone or can be combined. In addition to planar images, SPECT or SPECT/CT can also be implemented to increase the sensitivity of the method and offer more precise localisation of findings in the 3D space [4].

The early MIBI, late MIBI and TcO4 thyroid scan images are mandatory to detect and localise abnormal PGs preoperatively. Human intervention is, hence, irreplaceable. Artificial intelligence and, more specifically, image detection methods, can assist medical experts and reduce the workload in the everyday routine of medics [5]. Deep learning methods [6], which have received much attention recently [7,8], can offer timely and accurate solutions for several medical imaging tasks. The emergence of large-scale image datasets catalysed the development of deep convolutional neural networks (CNN) [9], which specialise in object detection, segmentation, and classification tasks.

The present study employs an innovative CNN topology for analysing the three associated images simultaneously to perform first-level discrimination between patients with PGs and patients with normal PGs.

## 2. Related Work

Current research has focused on PG recognition during surgery, using novel optical technologies, which are accompanied by DL algorithms to process real-time videos and images and localise PGs. However, there is no related work that addresses the issue of PG detection from scintigraphic images performed with ^99m^Tc-sestamibi (MIBI), which is a very common strategy for the preoperative examination of hyperparathyroidism. Here, we describe related works that employ artificial intelligence methods for detecting PGs, multiglandular disease (MGD), parathyroid adenomas (PTA), and hyperparathyroidism (HPPT) from disparate image sources; such methods deliver their results preoperatively.

Sandqvist et al. [10] proposed an ensemble of decision trees with Bayesian hyperparameter optimisation for predicting the presence of overlooked parathyroid adenomas (PTA) at a preoperative level using ^99m^Tc-sestamibi-SPECT/CT technology in multiglandular disease (MGD) patients. The authors used six predictors, namely the preoperative plasma concentrations of parathyroid hormone, total calcium and thyroid stimulating hormone, the serum concentration of ionised calcium, 24-h urine calcium, and the histopathological weight of the localised PTA at imaging. The retrospective study involved 349 patients, whilst the dataset was split into 70% for training and 30% for testing. The authors designed their framework utilising two response classes; patients with the single-gland disease (SGD) correctly localised at imaging, and MGD patients in which only one PTA was localised on imaging. Their algorithm achieved a 72% true-positive prediction rate for MGD patients and a misclassification rate of 6% for SGD patients. This study confirmed that AI could aid in identifying patients with MGD for whom ^99m^Tc-sestamibi-SPECT/CT failed to visualise all PTAs.

Yoshida et al. [11] employed RetinaNet [12], a DL network, for the detection of parathyroid adenomas by parathyroid scintigraphy with 99m-technetium sestamibi (^99m^Tc-MIBI) before surgery. The study enrolled 237 patients, which underwent parathyroid scintigrams using ^99m^Tc-MIBI, and determined them to be positive or negative cases. Those patients’ scans included 948 scintigraphs with 660 annotations, which were used for training and validation purposes. The test set included 44 patients (176 scintigrams and 120 annotations). The models’ lesion-based sensitivity and mean false-positive indications per image (mFPI) were assessed with the test dataset. The model yielded a sensitivity of 82%, with an mFPI of 0.44 for the scintigrams of the early-phase model. For the delayed-phase model, the results reported 83% sensitivity and 0.31 mFPI.

Somnay et al. [13] employed several ML models for recognising primary hyperparathyroidism using clinical predictors, such as age, sex, and serum levels of preoperative calcium, phosphate, parathyroid hormone, vitamin D, and creatinine. The study enrolled 11,830 patients managed operatively and included three high-volume endocrine surgeries. Under a 10-fold cross-validation procedure, the Bayesian network was superior to the rest of the ML models, achieving 95.2% accuracy and an AUC score of 0.989.

Imbus et al. [14] benchmarked ML classifiers for predicting multiglandular disease (MGD) in primary hyperparathyroidism patients. The study involved 2010 participants (1532 patients with a single adenoma and 478 with MGD). The 14 predictor variables included patient demographic, clinical, and laboratory attributes. The boosted tree classifier was found superior to the rest ML modes, reaching an accuracy of 94.1%, a sensitivity of 94.1%, a specificity of 83.8%, a PPV of 94.1%, and an AUC score of 0.984.

Chen et al. [15] applied transfer learning for the automatic detection of HPPT from ultrasound images annotated by senior radiologists. The study involved 1000 ultrasound images containing HPPTs, of which 200 images were used to evaluate the developed model. For this purpose, they employed three well-established convolutional neural networks toanalyse the HPPT ultrasound and suggested potential features underlying the presence of HPPT. They achieved a recall maximum of 0.956.

To the best of our knowledge, there is no other work in the preoperative recognition of parathyroid glands using scintigraphy with ^99m^Tc-sestamibi (MIBI).

## 3. Materials and Methods

### 3.1. Dataset and Data Processing

From January 2010 to December 2019, 632 patients with HPPT were referred to our department for parathyroid scintigraphy. 607 of these patients had biochemical evidence of primary HPPT, and 25 had refractory secondary or tertiary HPPT, due to end-stage renal failure. The dataset is detailed in Table 1. We used the planar dual-phase technique in all patients, and additionally, whenever judged necessary by the medical experts, the thyroid subtraction technique. ^99m^Tc-pertechnetate (TcO4) for thyroid delineation was administered either after the conclusion of the dual-phase study or on another day. For planar imaging, we used a pinhole collimator placed 10 cm over the neck.

A SPECT/CT imaging session focused on the neck and the mediastinum through the use of a high-sensitivity parallel-hole collimator took place approximately 30 min post tracer injection. However, only planar imaging data have been included in the present study. Planar and SPECT/CT imaging were performed using the Hawkey-4 system (GE Healthcare). Two senior medical experts retrospectively evaluated the planar scintigraphic studies. In a few ambiguous cases, the final decision was achieved by consensus.

### 3.2. Data Processing and Augmentation

**Figure 1:**
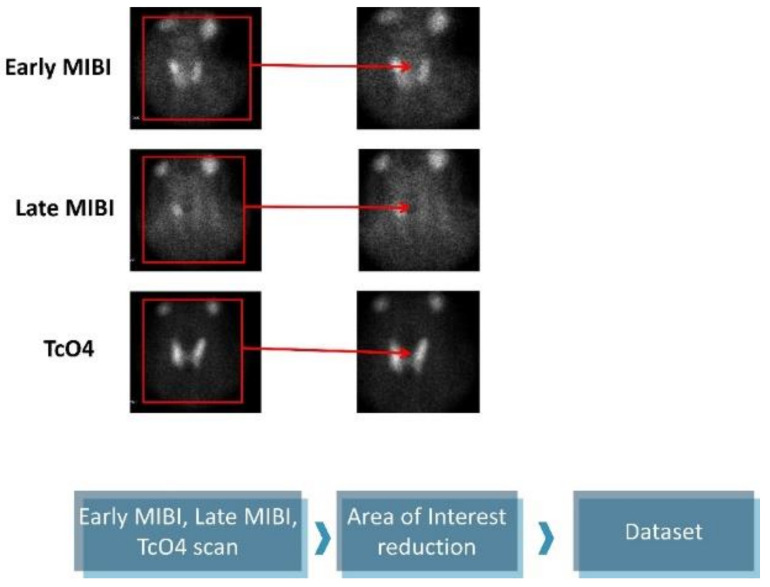
Data preprocessing.

The original images are 1400 × 1050 pixels and contain five subfigures. The informative details were gathered from the early MIBI, late MIBI, and thyroid TcO4 images. Next, the annotations and the irrelevant artefacts were removed by reducing the area of attention, as presented in Figure 1. The final images are 350 × 350 pixels in jpeg format. Data preprocessing was performed using the OpenCV library, written for the Python programming language.

Online augmentations took place during the training phase of each iteration. Data augmentation is a mandatory method to increase the generalisation capability of the proposed CNN approach and avoid overfitting. Data augmentation ensures the model becomes robust to slight local variations in the findings. In this experiment, we avoid strong data augmentation methods that distort the initial image completely. The parameters of applied augmentations are presented in Table 2.

### 3.3. Multipath Virtual Geometry Group Network (ParaNet)

#### 3.3.1. Virtual Geometry Group Component

Instead of developing a new CNN architecture from scratch, which is the traditional strategy in deep learning techniques [16], and its results highly depend on the dataset’s size and quality, an alternative method, called transfer learning, was preferred. With transfer learning [17], it is possible to exploit the knowledge of a specific CNN, which has been trained on a specific domain, to make predictions for another domain. The CNN developed by the Visual Geometry Group (VGG) [18] is widely utilised for medical image classification tasks. Thus, employing VGG was preferred for the experiments in this work.

During the process of pretraining using the ImageNet dataset [19], VGG has learned to extract task-specific image features. Hence, the model’s weight values are predefined by the domain it has been trained on. However, to allow problem-specific feature extraction, some of the layers are designed to be trainable, i.e., to learn from the input images of the present task. We select the architecture of VGG19 as the main component of ParaNet, due to recent success of VGG19 in related tasks [7,8,20].

VGG19 includes two convolutional layers of 64 filters, followed by another two convolutional blocks of 128 filters. Those two blocks are responsible for extracting well-visualised image features, such as edges and shapes of the image findings. These two groups are followed by a three-layer convolutional block of 256 filters per convolutional layer. Finally, another three-layer block of 512 filters per layer is applied. The latter groups extract more abstract and decisive deep image features that integrate local and global patterns of interest.

We employ the parameters and the setup of a modification of VGG19, called FF-VGG19, which has been introduced by the present team of authors for related tasks [20,21]. This CNN is a VGG19-based modification that allows for feature fusion (FF) by connecting the convolutional blocks with each other. Hence, the hierarchical nature of VGG19 is no longer a constraint. This modification has proven to be more efficient because it allows for more important features to be extracted during the training process.

#### 3.3.2. Parathyroid Network (ParaNet)

**Figure 2:**
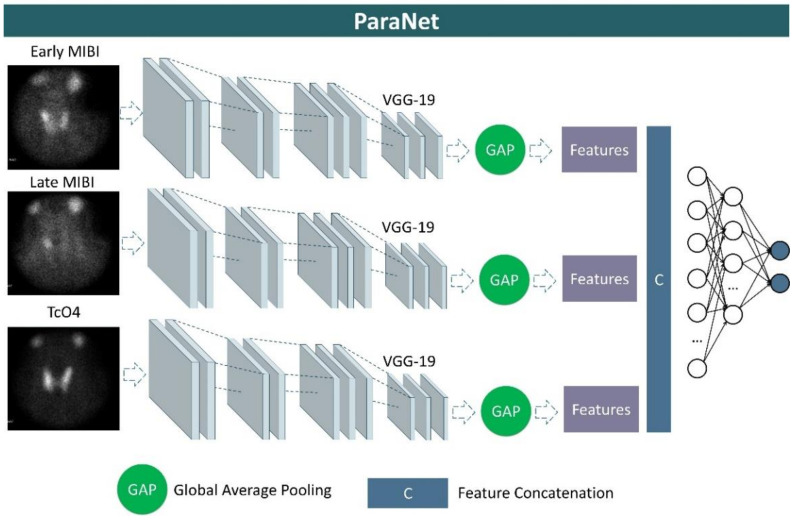
Graphical representation of ParaNet.

We developed a three-path CNN based on the modified VGG19 component discussed above. Each path is identical and is responsible for processing one of the three images associated with each patient’s case. More specifically, at the top of each VGG19 component, a global average pooling layer is applied, followed by a concatenation layer, which unites the extracted features from the early-phase image, the late-phase image, and the thyroid scan image. A densely connected network (classifier) is applied after the concatenation layer to distinguish between relevant and irrelevant image features. This network contains the input layer, which is connected to the output of the global average pooling layer, two hidden layers of 1500 and 750 units (neurons), and an output layer of two nodes, which represents the two classes, and is activated by the Softmax function. A graphical representation of ParaNet is illustrated in Figure 2.

### 3.4. Experimental Setup

A 10-fold stratified cross-validation procedure was followed. Stratified cross-validation split the complete dataset into ten non-overlapping folds. During each iteration (10 in total), a unique fold was used for evaluation, whereas the remaining folds constitute the training sets. The stratified validation ensures class-aware creation of these folds to avoid biased sets. At the end of the iterations, each fold of the original dataset was used for evaluation exactly once. The final metrics were calculated based on the average metrics of the ten iterations. The final confusion matrix is populated from each iteration.

ParaNet was trained for a maximum of 200 epochs. Early stopping was applied to avoid redundant training epochs. More specifically, the model instantly aborted the remaining training epochs when the training accuracy reached 98%. Each epoch of training was performed in mini-batches of 32 examples.

The agreement rating is reflected in the overall accuracy (ACC) score and in Cohen’s Kappa coefficient. However, considering medical expertise as the ground truth, the total number of true-positive (TP), true-negative (TN), false-positive (FP), and false-negative (FN) samples was recorded. The model was also evaluated using the corresponding sensitivity, specificity, positive predicting value (PPV), negative predicting value (NPV), F1 score, and area under the curve score (AUC).

**Figure 3:**
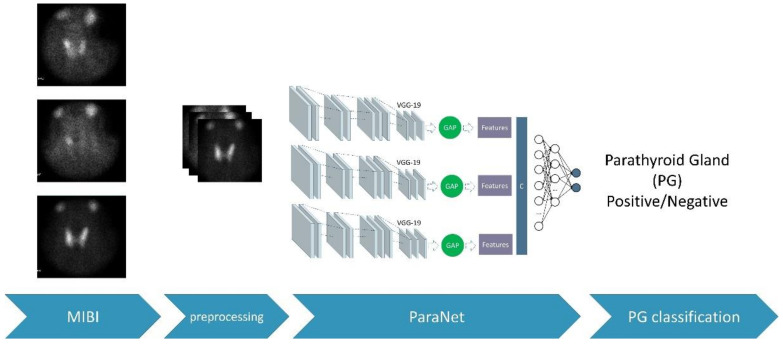
The overall process.

During each fold, the discussed metrics were recorded. These metrics are based on the number of true-positive, false-positive, true-negative, and false-negative subjects of the fold. After the 10th fold, the average values of the above metrics were recorded, and the final confusion matrix was extracted. The complete experimental procedure is presented in Figure 3.

## 4. Results

The dual-phase technique was applied to all patients of the study. The TcO4 thyroid scan was additionally performed in 514 cases. Medical experts identified one or more abnormal PGs in 414 scans. In the remaining, no abnormal findings could be disclosed either with the dual-phase or the thyroid subtraction technique.

### 4.1. Classification Results

ParaNet yielded optimal performance, successfully identifying 399 abnormal scans (aPG, true-positives) and 163 nPG scans (true-negatives). The total false-positive predictions were reported to be equal to five, whilst the false-negative predictions were found to be 15. These results are summarised in the confusion matrix presented in Table 3.

ParaNet exhibited a top performance, reaching an accuracy of 96.56% in distinguishing between PG and nPG scans. Its sensitivity and specificity were 96.38% and 97.02%, respectively. The PPV and NPV values were 98.76% and 91.57%, respectively. Finally, the F1 score was 97.56%, which indicates that, despite the imbalance issue, the model is not biased.

The following results demonstrate the FF-VGG19 component of ParaNet’s efficiency. Replacing the FF-VVG19 network with similar state-of-the-art CNNs yielded suboptimal results, as reported in Table 4.

It is also demonstrated that all VGG variations performed better than the rest of the models. However, statistical significance tests are required to determine whether these discrepancies are of major importance.

### 4.2. Ablation Study

An auxiliary set of 50 scans was held back from the initial training and evaluation dataset. This set includes patients that underwent parathyroidectomy. Hence, the assignment of the labels of each scan was confirmed by surgery. Scans with abnormal PG findings were reported to be equal to 38, whereas 12 scans were classified as normal.

ParaNet was trained with the complete initial dataset of 582 scans under the same parameter and hyperparameter setup for this experiment. ParaNet was deployed to predict the labels of the ablation study, producing the confusion matrix of Table 5.

ParaNet exhibited very promising results for the unseen data of the ablation study. A slight decrease in accuracy was observed (92%). The model’s sensitivity was 94.74%, and the specificity was 83.33%. The PPV was found to be 94.74%, whilst the NPV was 83.33%. The F1 score is reported to be 94.74%.

The predicted labels of ParaNet were also compared to the experts’ diagnostic yield. With reference to the parathyroidectomy results, the accuracy of the medical experts reached 94% (97.37% sensitivity, 83.33% specificity). The experts’ diagnostic yield was slightly better (by 2%). A case-to-case comparison is presented in Table 6. The agreement between the model and the experts was 96% (Cohen’s Kappa coefficient of 0.88).

## 5. Discussion

The results of the present study demonstrate that PG detection from parathyroid scintigraphy with ^99m^Tc-sestamibi (MIBI) is plausible. The proposed ParaNet, a multipath VGG19-based CNN, can discriminate between normal and abnormal scans with an accuracy of 96.56% under a 10-fold cross-validation procedure.

The study has some limitations. Firstly, ParaNet was evaluated based on its performance in distinguishing normal from abnormal scintigraphic scans. Although undeniably useful as a first step, localisation of the abnormal findings (PGs) is necessary for a medical imaging technique to serve as an assisting tool for medical experts. Detection and segmentation of abnormal PGs could be achieved in the future by employing two methods. Given a proper annotation of the dataset, object detection models can be developed, such as the YOLO network or similar state-of-the-art CNNs. This requires a lot of effort regarding the annotation of the scintigraphic scans, which will serve as the training set. Moreover, such a method is applicable and would yield promising results given a larger availability of annotated data. A second approach is to integrate recent explainability algorithms, such as the Grad-CAM [22] algorithm and its derivatives (Grad-CAM++, etc.) to visualise the areas where the model seeks decisive features that aid in the discrimination task. This method would, hopefully, reveal abnormal PGs located in the scans. This method is non-trivial, since the characterisation of the PG abnormality is based on the comparisons between the three associated images. Hence, the model may actually locate an interesting finding, but its assessment requires some reasoning.

Except for localising the PGs inside the image, the model lacks any informativeness regarding the type of findings. For example, findings corresponding to multiglandular disease are not reported separately from the others. The same applies to the location of the findings, e.g., ectopic/intrathyroidal. A further limitation of the study is the scarcity of surgically-confirmed image sets. Hence, training and model evaluation were performed with reference to the medical experts’ scintigraphy interpretation. The ablation study, which employed 50 patients that underwent parathyroidectomy, demonstrated a slight performance decline. Although the accuracy of ParaNet remained at a top level (92%), the small decline cannot be overlooked.

Traditional detection methods involve human intervention. Experienced medical experts analyse the three images to achieve the diagnosis. This procedure can be time-consuming, especially for new medical staff. The proposed model can operate either as a part of an embedded framework in the medical image acquisition device or standalone. Once the model is trained, it can produce a diagnosis in no more than a second. As a result, it is suitable for online analysis. Moreover, a trained DL model is lightweight, and the file size does not exceed 200 mb. Therefore, it is also transferable.

This study broadens the horizons of future research opportunities to improve the diagnostic performance of ParaNet and enhance its explainability. Future research should consider the incorporation of clinical and demographic predictors into the final decision, which could serve as auxiliary inputs and improve performance. Furthermore, this methodology can be applied to other imaging modalities, such as the ultrasound test. With the lack of ultrasound image datasets, comparison between the presented and the ultrasound methods is impossible.

## 6. Conclusions

This study explored the efficiency of deep learning in distinguishing scans with abnormal PGs using scintigraphy with ^99m^Tc-sestamibi (MIBI). Three images from each patient were considered the input of the model. Early MIBI, late MIBI, and thyroid scans were processed simultaneously by a three-path VGG19-based network called ParaNet. The results are very promising (96.56% accuracy, 96.38% sensitivity, 97.02% specificity). This study broadens the horizons of future research opportunities to improve the diagnostic performance of ParaNet and enhance its explainability. The proposed network is the first to introduce automatic discrimination of PG and nPG scans acquired by scintigraphy with ^99m^Tc-sestamibi (MIBI). This methodology could be applied to the everyday routine of medical experts for real-time evaluation or educational purposes.

## Figures and Tables

**Table 1:** Characteristics of the study’s dataset.

Information	Value
Date	2010–2019
Total Number of Subjects	632
Male Subjects	21%
Female Subjects	79%
Average Age	57.2 years
Primary HPPT	607
Secondary/Tertiary HPPT	25

**Table 2:** Data augmentation parameters.

Transformation	Degree of Freedom
Rotation	±20 degrees
Sheer	±0.1
Flip	Horizontal

**Table 3:** Confusion matrix.

Confusion Matrix		Reference Label	
		aPG	nPG
Predicted Label	aPG	399	5
	nPG	15	163

aPG, scans with abnormal PGs; nPG, negative studies.

**Table 4:** Comparison of ParaNet performance when using different CNN components for each path. ACC, Accuracy; SEN, sensitivity; SPE, specificity; PPV, positive predictive value; NPV, negative predictive value; F1, F1 score.

CNN	ACC (%)	SEN (%)	SPE (%)	PPV (%)	NPV (%)	F1 (%)
FF-VGG19	96.56	96.38	97.02	98.76	91.57	97.56
VGG19	93.47	94.44	91.07	96.31	86.93	95.37
VGG16	92.61	93.00	91.67	96.49	84.15	94.71
MobileNet	93.47	94.20	91.67	96.53	86.52	95.35
Inception V3	90.21	90.58	89.29	95.42	79.37	92.94
Xception	92.27	93.24	89.88	95.78	84.36	94.49
EfficientNet	87.80	87.68	88.10	94.78	74.37	91.09
DenseNet	79.55	77.54	84.52	92.51	60.43	84.36
ResNet	70.27	69.57	72.02	85.97	48.99	76.90

**Table 5:** Confusion matrix of the ablation study.

Confusion Matrix		Reference Label	
		aPG	nPG
Predicted Label	aPG	36	2
	nPG	2	10

**Table 6:** Case-to-case comparison between the medical experts’ diagnostic yield and ParaNet for 50 parathyroidectomy-confirmed scans.

Confusion Matrix		Nuclear Medicine Expert	
		aPG	nPG
Predicted Label	aPG	38	1
	nPG	1	10

## Data Availability

The dataset is available upon a reasonable request and is not publicly released for ethical reasons.
